# The Role of Adult Day Services in Supporting the Occupational Participation of People with Dementia and Their Carers: An Integrative Review

**DOI:** 10.3390/healthcare6020043

**Published:** 2018-05-08

**Authors:** Janice Du Preez, Jeannine Millsteed, Ruth Marquis, Janet Richmond

**Affiliations:** School of Medical and Health Sciences, Edith Cowan University, Joondalup 6027, Australia; j.millsteed@ecu.edu.au (J.M.); r.marquis@ecu.edu.au (R.M.); j.richmond@ecu.edu.au (J.R.)

**Keywords:** adult day services, adult day care center, community-based services, dementia, occupation, primary carers

## Abstract

The increasing numbers of people with dementia places considerable stress on health and aged care services and has resulted in the development of community adult day services. **Aim**: The aim of this integrative review is to determine the extent to which these services support the occupational participation of people with dementia, and how they impact their primary carers. **Method**: The mixed-methods appraisal tool (MMAT) was used to identify relevant studies in the period 2011–2016. **Results**: Nine databases were searched and yielded 16 articles with a variety of research designs for inclusion in the review. **Conclusions**: Findings indicate that adult day services use a range of approaches to support attendees and their carers. In spite of these efforts, there appears to be a lack of interest in utilizing these services while a person is in the early stages of dementia. This suggests that policies in aged care, such as aging-in-place, need to consider the pressure and stress they exert on carer’s quality of life. Another consideration is to better promote the benefits of participating in adult day services in the early stages of dementia for both the attendees and their carers, thereby delaying the tendency towards early institutionalization.

## 1. Introduction

### 1.1. Aim of This Review

The aim of this integrative review is to assess current research relating to the role adult day services play in the occupational participation of people with dementia. The study also explores if and how adult day services impact primary carers. Existing research was considered, relevant studies were evaluated, and implications for practice and future research are discussed.

### 1.2. Occupation

Humans have an innate drive to accomplish and produce through active participation in occupational pursuits [1]. This motivation for competency through occupational engagement enables a sense of mastery which in turn re-enforces a sense of “self” or personal identity [2,3]. Occupational therapy practitioners are concerned with understanding the specific social and physical contexts within which people want to pursue occupations [4]. Human occupation includes the following: activities of daily living such as self-care and self-maintenance; play, which includes leisure and recreation; and instrumental activities of daily living that provide services to others such as caring for family through productive employment [5,6]. Occupations are that which humans do, why and how they do them and what they think and feel about them as they occur in the context of time, space, society and culture [2]. Research suggests that the presence of impairment may or may not impact well-being. The determining factor has been identified as the effect an impairment has on occupational participation [7].

### 1.3. Occupational Participation

Occupational participation provides opportunities for inclusion and involvement with others, through activities that have meaning and significance both personally and socially [1,8]. Occupational performance relates to doing a task associated with occupational participation, such as how well an individual is able to plan and execute a lesson plan when teaching English [8]. 

Engagement in occupations of interest has a positive impact on cognitive function [2,3]. However, cognitive ability can worsen significantly with the onset of dementia undermining the ability to participate [9,10].

### 1.4. Background

The number of older Australians using residential aged care services during the course of a year rose by 36% in 2010–2011 compared to services accessed during 2002–2003. However, the majority of older Australians (71%) did not access residential aged care services in 2010–2011 with many preferring to remain at home for as long as possible [11]. With nearly 71% of older adults living at home, unpaid informal care provided by relatives and friends underpins the community services system in Australia [11].

The crucial role of informal caring is commonplace with an estimated 1.2 million people being primary carers who provide spousal care of a person with dementia in Australia. In 2012, 38.9% of primary carers reported spending 40 h or more per week caring. The replacement value of unpaid care provided in 2014 cost above $1 billion per week. Australia depends on unpaid, informal spousal and intergenerational carers [12,13]. An escalation in female workforce participation, an aging population and an aging caring population means that Australia will be faced with a shortage of more than 150,000 paid and unpaid carers for people with dementia by 2029 [14,15,16].

Quality of life is typically represented as good health, longevity, and functioning. This holistic view underpins the World Health Organization’s (2001) *International Classification of Functioning, Disability and Health* policy. The WHO (2014) describes positive mental health as:

“… a state of well-being in which the individual realizes his or her own abilities, can cope with the normal stresses of life, can work productively and fruitfully, and is able to make a contribution to his or her community”.

Good health is an important resource as it influences participation in many aspects of life. However, what constitutes good quality of life differs from person to person and is the summation of personal preferences, circumstances, environments, social and cultural norms [17].

Quality of life is increasingly being recognized as a combination of factors relating to physical, mental and social wellbeing by health care professionals, researchers, policymakers and the public. These factors can include levels of independent functioning, support networks, economic status, an individual’s perceptions and beliefs, policies and inequalities. Psychological distress can cause considerable suffering and may contribute to people experiencing higher mortality rates, social isolation and poor quality of life [18]. An effective response to mental health issues is to improve access to services and support and promote environmental, social and economic participation for individuals, their families, and carers [17,19].

Vreugdenhil argues that promotion of the aging-in-place approach by the Australian aged-care sector may mean an increased reliance on informal carers. The contradiction between policies and the assumption that employed informal carers are willing to provide unpaid care causes distress in informal carers. Therefore, the impact of the choice to take on the caring role needs further investigation and consideration when making policy decisions.

Risk factors for carer stress include living with the person with dementia, previous health issues, the age, personality and coping style of the carer [13,20,21]. Primary carers who are family of the person with dementia, tend to experience higher stress levels with 30% of carers reporting having depression compared to those who cared for people with other health conditions and non-carers. Primary carers who care for those who live with dementia tend to be greatly impacted by the caring role as they provide longer hours of care daily and 68% reportedly have frequent sleep disturbances due to their caring role [16,20].

A survey conducted by Jowsey, McRae, Gillespie, Banfield, and Yen [22] revealed that 96.2% of carers suffered from chronic illness themselves and that those with co-morbidities were those who had the highest levels of care responsibility. Having health problems concerns older spousal carers as they worry about the impact of their illness on their care recipient and whether or not this could cause them to be separated as a couple. Similarly, adult children caring for their father or mother report being concerned about the consequences of their illness on their parents [21]. Policies that consider infrastructures that support carers, enabling them to care for themselves, have the potential to improve caregivers’ quality of life and health outcomes.

Vrkljan, Leuty and Law found that a common concern raised by each of their study participants was the fear of being required to move into institutional care. A person’s level of independence is closely linked to their ability to independently perform instrumental activities of daily living (IADLs) such as preparing a meal, shopping for groceries, budgeting, being medication compliant or doing electronic banking. While people with dementia retain enough cognition to perform personal care-related activities, dysfunction in IADL performance is often overlooked. Problems with performance are associated with reduced self-efficacy, poor quality of life, increased carer burden, a need for increased services and ultimately a rise in health care costs [23,24,25].

Adult day services in Australia provide multilingual and culturally diverse services to community-dwelling older adults with physical, intellectual and mental disabilities. Service facilities are generally socially orientated with service delivery aimed at reducing psychosocial problems, ensuring maintenance or recovery of performance of activities of daily living and offering a broad range of group activities to enhance socialization. These services generally operate during daytime hours, Monday through Friday. Some staffing complements include allied health professionals such as physiotherapists and occupational therapists, while other services may employ a leisure coordinator and personal care assistants [19]. Service delivery is personalized and adapts as client’s cognition and or health status deteriorates [26].

Adult day service attendance has been found to positively impact physical and psychological functioning by reducing feelings of social isolation, anxiety, and depression. Attendance is also associated with fewer behavioral and sleep problems and improved quality of life for both the attendees and their caregivers, lowered stress levels for dementia caregivers and fewer behavior and sleep problems in care recipients [27,28,29,30,31].

Tse and Howie found that dissatisfaction with some aspects of adult day services such as the food and opportunities to engage in meaningful occupational participation was a common theme among participants, with one participant commenting that the activities were “childlike” (p. 138). The researchers stated that structure and activity content of adult day services was largely dependent upon the skills and attitudes of staff and that occupational participation was dependent upon age-appropriate, meaningful and purpose directed activities. They further argued that engagement in a meaningful activity provided more than satisfaction, it gave attendees a sense of purpose which enhanced their quality of life [32].

Evidence suggests that the efficacy of adult day services depends upon user attendance, programming and caregiver support [33,34] and is reflected in improved mood, functioning and reduced carer burden [35].

Research focuses on caregiver well-being rather than on whether adult day services influence attendee outcomes or not [36,37]. Additionally, insufficient attention has been paid to the impact of adult day service use on the quality of life of attendees [38]. The aim of this review is to extend understanding of how attendance at adult day services supports attendees with dementia their primary carers. Understanding these factors may inform service providers in their efforts to develop best practices during program planning. Additionally, areas requiring future research will be identified [39,40].

This review investigated literature between the period 2011 to 2016. The primary focus of the review was to explore literature that identified the role attendance at community-based adult day services play upon the occupational participation of attendees with dementia and that of their primary carers. It aimed to build on the work by Fields, Anderson and Dabelko-Schoeny [36], Gaugler [37,41], Gitlin [42] and Zarit [43].

## 2. Method

The nine databases included in the search were: PubMed, Web of Science, Medical Database (ProQuest), Medline, BMJ Best Practice, Scopus, PsycINFO, OTSeeker, CINHAL. The search was conducted between May 2016 and November 2016. The keywords searched included ‘adult day services’ OR ‘community-based adult day services’ OR ‘adult day centers’ OR ‘dementia’ OR ‘occupation’ AND ‘carers’.

The mixed methods appraisal tool (MMAT) was used to assess the quality of studies [44]. Papers met the inclusion criteria if they reported on adults over the age of 65 with (diagnosed/identified) Dementia or Alzheimer’s disease, were peer-reviewed and published in English between 2011 and 2016 with abstracts that met the aims of the review. Once identified and cleared of duplicates the studies were reviewed in full to identify their objectives and results.

## 3. Results

Application of the MMAT process identified 16 studies for inclusion in the integrated review (Table 1). Various research designs were represented across these studies, including qualitative studies (*n* = 6), quantitative non-randomized studies (*n* = 3), mixed-method design studies (*n* = 4), a systematic review 35 studies, an integrated review 19 studies and a narrative review of 76 studies informed this review. No randomized controlled studies were found. All sixteen studies reviewed included both genders in the sample size. One study failed to report the participant sample size.

### 3.1. Key Findings

People living with dementia and their primary carers are at risk of further decline, social isolation, increased anxiety, and depression while the current health care system has little to offer to mitigate these issues.

Adult day services were established to meet the growing need of frail elderly people with or without dementia who choose to stay at home and to provide respite for families who provide daily care to relatives. These services aim to enhance the occupational participation, quality of life and well-being of attendees and to support older adults who choose to remain in their own homes for as long as possible.

### 3.2. Reasons for Adult Day Service Utilization

Reasons for utilization of adult day services ranged from people with dementia’s need to socialize to the need for respite from the caring role for carers [45]. Adult day services attendance has been found to positively impact physical and psychological functioning by reducing feelings of social isolation, anxiety and depression with attendees with early-stage dementia reporting that participation in a social group had a positive impact on them [45,46,47]. This was evidenced by their improved mood. Their experiences are further enhanced by newly formed friendships with peers with whom they feel comfortable, understood and therefore safe. Additionally, attendees reported having fun and feeling validated. Staff and carers reported that their care recipients seemed happier, that they were more socially engaged and that relationships within the home improved as a result of their attendance.

Carers reported increased engagement with their care recipients upon returning from adult day service attendance. This was due to their improved mood and them sharing interesting events that occurred throughout the day. This positive outcome was perceived as the reason for prolonged aging-in-place by staff and carers alike [45,47].

Family carers have little to no contact with the adult day service other than to ready their care recipient for the day’s attendance and have little knowledge of how their care recipient spends their time while attending adult day service [45]. Adult day services that offer comprehensive services that engage dementia caregivers by way of phone calls or one-on-one carer meetings to address their areas of concern, invite carer collaboration in planning meetings, provide carer education, counseling and case management were seen to facilitate increased service utilization and delay early institutionalization [35,45,47,48,49].

### 3.3. Program Planning

For most people living with dementia, their goal is to live a good quality life filled with fun, laughter and friendships. This is an important consideration for program planners who often make improved mood and functional performance their goal. Staff often have to be flexible and take on multifaceted care roles as they attempt to meet attendee preferences and needs, and family carers expectations [45,47].

Adult day services’ ability or inability to offer a variety of activities that were person-centered and held meaning for attendees was a motivating factor for use. Additionally, it is important to find ways in which people with dementia can make a meaningful contribution. Participation was also dependent upon adult day services having the resources to offer one-on-one interventions with attendees who were cognitively impaired [45,47].

### 3.4. Benefits of Use

The benefits of adult day service use for people with dementia are perceived by their carers to be opportunities for social interaction and occupational participation [50,51,52]. Regular attendance is also perceived by carers to provide their care recipients with a sense of structure and routine to their daily lives [46,49].

A study to evaluate participant and caregiver outcomes by Logsdon, Pike, Korte, and Goehring found that 6 months after attendance at an adult day service had begun, attendees exhibited significantly less depression and behavioral issues compared to participants who did not attend an adult day service. Furthermore, findings show that female spousal carers had higher levels of depression than adult daughters who cared for a parent with dementia who attended and adult day service.

Additionally, female spousal cares of non-attendees were more likely to opt for earlier admission to an aged care facility than those whose care recipients attended an adult day service. Findings further suggest that consistency and higher attendance levels are associated with systematically improved caregiver outcomes. According to Zarit, et al. this result is significant as it supports the notion that adult day service attendance provides time apart and relief from care-related stressors and may mitigate the negative impact associated with sustained exposure to stress. Additionally, caregivers of attendees exhibit less stress about their care recipient’s mood, memory loss and changes in behavior [35,52,53,54].

### 3.5. Reasons for Under-Utilization of Adult Day Services

Adult day service use can assist with relieving carer stress and delay institutionalization of the person with dementia [55]. Consumer-directed care policies that promote aging-in-place for older adults living in the community have increased the burden of self-managed care for those carers. Whilst evidence supports the benefits of adult day service use for caregivers, utilization by those caring for people with dementia is negligible with 89% of primary carers never having used respite care. Aside from the lack of access to services, research suggests that inflexible service availability may be the reason for limited use [16,49,50,52,55].

Lloyd and Stirling argue that some carers who engage with adult day services experience unintended negative effects, resulting in confusion and a perceived loss of control, fueling their reluctance to use services. Factors such as concern for privacy violation and cost of service provision diminishes use of services by carers. Furthermore, interaction with adult day service providers requires carers to adopt institutional processes and intrudes upon their care recipient’s privacy, albeit it to promote that person’s quality of life. Additionally, imperatives of service delivery systems place carers in a vulnerable position seemingly prioritizing these systems above the needs of caregivers [55].

Donath, Winkler, Graessel and Luttenberger explored this issue of non-utilization and found that the level of support offered by an adult day service predicts utilization and that carers require the provision of a strength-based, person-centered activity program as a condition for use. Additionally, carers expect their care recipients to be treated with care, respect, and dignity while attending an adult day service [50,51]. Utilization of services is often determined by effective referral by health professionals. Medical practitioners were identified as having limited knowledge of community support services and access to information resulting in poor referral processes and therefore, poor utilization by family carers and people living with dementia [49].

Affordability is an issue for some carers who constantly weigh their care recipients needs against the economic burden of meeting those need. Carers reported having difficulty assisting their care recipient with their activities of daily living in preparation for the day’s attendance. This was exacerbated by their care recipient’s co-morbidities and resistance to attendance.

Transport was reported to be a significant barrier to utilization for carers. Use of public transport to access adult day service as opposed to transport being provided was found to be difficult and time-consuming [45].

Another barrier to use is attendance being conditional upon attendees being able to independently self-care. As attendees experience a decline in functional performance due to disease progression, they are no longer able to attend to their self-care needs independently [47].

Overcoming carer stoicism, feelings of guilt and perceptions of societal disfavor at their failure to care when utilizing adult day services, may require carer education on the effect that accumulated stress can have on them physically and psychologically. In order to allay feelings of guilt in using a service, family caregivers need reassurance that their care-recipient will be safe and have opportunities for social engagement during attendance [50,51,52]. This would see carers utilizing respite adult day services sooner, which will allow them time for relief from their caring role and provide opportunities for self-care [49,52].

In order for staff to attend to attendees with cognitive impairment, they are sometimes seated separately during meal times and group activities [45]. Whilst separation may lead to stigmatization, group inclusion for attendees with cognitive decline appeared to promote their disengagement from activities requiring cognitive demand. However, Gaugler found staff overcame these issues through validation and one-to-one interaction. Phinney and Moody argue that community-based services that endeavor to minimize stigma and normalize attendee’s experiences, rather than focus on symptom management, may attract greater utilization.

People with dementia and family carers require access to flexible, informative and collaborative services [21,26]. The *Carer Recognition Act 2004* provides recognition of carers and requires all government-funded service providers to comply with the Carers Charter. This includes working collaboratively with carers, or persons that represent carers in developing policies or programs and in strategic or operational planning that might impact upon carers in their caring role.

A current trend in service delivery is towards consumer participation. Studies advocate for policy reform or treatment protocol design which includes input from those that have to live with the recommendations, and who will be receiving care [21,26]. Service providers, in attempting to customize service provision, require an understanding of the individual’s perceptions and needs and of their experiences of multiple interventions in order to create opportunities for interactional co-creation of intervention design [26].

Adult day service staff and consumers reported their frustration at the lack of interest shown by community-based older adults in utilizing adult day services. It was thought the solution lay in more effective marketing strategies that promote the adult day service programs making communities more aware of the support available to them [45,50]. Donath et al. [56] contend that service providers need to be transparent and that carers should be informed of the advantages to attendance for both themselves and their care recipients.

### 3.6. Summary of Literature Review

People place high value on the opportunity to be self-sufficient and autonomous. Having the right to choose and implement preferred choices over everyday activities and the freedom to actively participate in society at all levels has been found to relate to quality of life with autonomy being the core value of person-centered care.

Research indicates that promotion of the aging-in-place approach by the aged-care sector may mean an increased reliance and expectation on informal carers to undertake the caring role, and that the impact of the choice to do so needs further investigation and consideration when making policy decisions.

Service providers, in attempting to customize service provision, require an understanding of the individual’s perceptions and needs and of their experiences of multiple interventions in order to create opportunities for interactional co-creation of intervention design [26]. An assessment of service needs should include questions that elicit primary carers’ perceptions of their levels of stress or depression and whether or not the carer feels the need for additional support.

Further evidence suggests that caregivers begin to utilize adult day services once the dementia has entered its late stage and the situation has begun to fragment. Intervention early in the caregiving process can offset carer stress through adult day service support, respite and education.

Increasing adult day service visibility, through media advertising in communities to target caregivers early, is desirable.

Findings from this review also suggest that the organizational ethos of adult day services impacts upon the service culture which determines the levels of utilization. A service culture that considers the social environment i.e., person-centered care, staff to attendee relationships, staff to staff relationships, staff to carer relationships and attendee peer group relationships as well as how the built environment may facilitate or inhibit attendee participation is an emerging area of research. This links directly with the paucity of research that explores the perspectives of people with dementia and provides justification for future research.

### 3.7. Methodological Strengths and Limitations

Systematic reviews and meta-analysis do not consider the impact of disease issues relating to care or treatment thereof [57]. An integrative review was identified as the most appropriate way to review theories and evidence and synthesize current literature that explores descriptive analysis of contextual detail [52,57]. The major limitation of studies reviewed was the small sample sizes and assessment had to be conducted with this limitation in mind. Commonalities in the general findings across studies highlight the need for further research in this area.

## 4. Conclusions

Adult day service attendance positively impacts attendees through their social engagement and participation in activities with peers with whom they feel safe and comfortable. Validation by adult day services staff was also shown to improve an attendee’s mood with carers evidencing this upon their care recipient’s arrival home.

Whilst evidence supports the benefits of adult day service use for primary carers, 89% of primary carers have never used respite care. Family carers have limited contact with the adult day service with little knowledge of how their care recipients are engaged throughout their day. This lack of knowledge coupled with service provider’s inability to offer person-centered activities, that hold meaning for attendees, contribute to non-utilization of adult day services. However, where service providers actively invited carer collaboration in program design, carer education, counseling and carer support, early institutionalization of people with dementia is delayed and is also associated with improved caregiver outcomes.

Evidence from Australian research is consistent with international research. Whilst there is a growing body of evidence on the subject of respite for caregivers both internationally and in Australia, there is a paucity of research that explores the perspectives of people with dementia and how they experience adult day services. Although available research addresses attendance at an adult day service, there is a substantial data gap relating to client and primary carer outcomes. Further research that explores the perceptions and occupational needs of people living with dementia and their primary carers whilst in the home and during adult day service attendance is needed.

## Figures and Tables

**Table 1:** Summary of studies included in the integrative review.

Authors	Aims	Participants	Methodology/Design	Findings	MMAT-Score
Low, Yap and Brodaty (2011)	To evaluate the outcomes of consumer-directed home and community care services for older adults.	N/A	A systematic review of 35 papers was conducted on home and community care services for older adults. MEDLINE, PsycINFO, CINAHL, AgeLine, Scopus and PubMed were searched from 1994 to May 2009. Results were reviewed independently by two researchers.	▪ Improved function and medication compliance increases with community service utilization and reduces early institutionalization	xxxx
Gustafsdottir (2011)	To explore different care approaches by family and staff, to understand how these might guide and enhance each other.	Family and staff and participant-observation	Qualitative research design Comprising 3 parts: (1) a longitudinal study of the family’s experience, (2) staff interviewed in groups (3) participant’s observation. The study commenced in 2003 and ended in 2009	▪ ADS attendance promotes routine activity in everyday life for the person living with dementia and encourages social interaction among peers ▪ ADS not only provide respite for carers and family members	xxx
Phinney and Moody (2011)	To evaluate a dementia-specific community-based social activities program tailored to meet the needs of people with early stage dementia.	(*N* = 10) interviews and 40 h of observation were conducted	Qualitative research design using interviews.	▪ People with early stage dementia have distinctive requirements ▪ Social activities meet the needs of this subset ▪ There are limited support options for this subset ▪ Effective community-based program development that meets the specific needs of people with early stage dementia is required ▪ Focusing on stigma reduction rather than on symptom management is desirable ▪ Program planning that is person-centered, fun and that promotes opportunities for meaningful contribution is important	xxx
Donath, Winkler, Graessel and Luttenberger (2011)	Examined how family caregivers are motivated to utilize community-based services.	(*N* = 404) family caregivers of people with dementia, of these (*N* = 128) were users of day care, (*N* = 269) were non-users and (*N* = 7) gave no details about utilization	A mixed-method study	▪ The age of the family caregivers and their perception of service provider’s effectiveness in caring for their care recipient are predictors of ADS utilization ▪ Family caregivers need more information regarding service provision and the advantages associated with accessing ADS ▪ Family caregivers anticipate that ADS include dementia-specific activities	xxx
Zarit, Kim, Femia, Almeida, Savla and Molenaar (2011)	Examined stressors associated with use of adult day services by people living with dementia on their family caregivers.	(*N* = 121) family caregivers whose care recipients attended an ADS	Quantitative research design using a within-person withdrawal design (A-B-A-B) compared how care-related stressors impacted upon carers on the day their care recipient attended an ADS to the days when they did not attend	▪ Care related stressors decreased on ADS days compared with non-ADS days ▪ Stressors decreased with ADS use as caregivers’ spent time apart from their care recipients	xxxx
Gill, White, and Cameron (2011)	To understand interactive experiences from the perspective of people dementia whilst attending an ADS.	(*N* = 22)	Qualitative study design using interviews	▪ People with dementia want to work collaboratively with services providers during the program planning stage ▪ Service providers need to create opportunities for collaborative planning	xxxx
Lloyd and Stirling (2011)	To explore the ‘ambiguous gains’ that family caregivers of people with dementia experience when accessing support services in the home.	(*N* = 33)	Qualitative study design using interviews from two independent projects	▪ Giving service providers access to the home may result in confusion on the part of the care recipient with dementia and perceived loss of autonomy for their family caregivers. ▪ Utilizing support services may highlight family carer’s sense of their inability to manage and adding to their feelings of guilt around increasing the governmental burden of care	xxxx
Robinson, Lea, Hemmings, Vosper, McCann, Weeding and Rumbles (2012)	To identify issues for family caregivers when accessing ADS.	(*N* = 27) (*N* = 10) carers whose family member refused to attend ADS And (*N* = 17) carers whose family member attended an ADS	A qualitative approach, utilizing telephone interviews, was adopted for this study which was conducted between August and December 2007 in Hobart, Tasmania.	▪ Australian family carers underutilize ADS ▪ Carers felt overwhelmed by information, the services provided and their car recipients safety while attending an ADS ▪ Carers were grateful for the time of respite ADS attendance affords them ▪ Better access to relevant information is needed	xxx
Anderson, Dabelko-Schoeny, Fields, and Carter (2012)	Studied how ADS provide services to people with dementia and the extent to which they support their caregivers. Staffing and facility characteristics and how they promoted service provision were also explored.	(*N* = 297) The data were from the 2010 MetLife National Study of Adult Day Services	Quantitative research design using logistic regression analysis	▪ ADS provided supportive services to family caregivers ▪ Multidisciplinary service provision was desirable as opposed to offering respite alone	xxxx
Gaugler (2014)	Examined why and how families and older adults utilize ADS.	(*N* = 26) 14 family members of clients and 12 staff members	Qualitative methodology using semi-structured interviews and observations	**ADS goals** ▪ Offer respite for families to provide daily care to relatives with dementia ▪ To enhance the functional independence, quality of life and well-being of attendees ▪ To support older adults who choose to remain in their own homes for as long as possible **ADS use** ▪ Low ADS utilization thought to be as a result of inadequate marketing strategies ▪ Affordability weighed against carer’s economic burden of care ▪ Staff have to be flexible and play multifaceted roles ▪ Carers reported increased engagement with their care recipients upon returning from adult day service attendance ▪ Adult day services who engaged family carers increased utilization	xxxx
Stirling, Dwan, and McKenzie (2014)	To explore carers’ expectations and perceptions of adult day respite services and their commitment to using services.	(*N* = 50)	A mixed-method case study approach	▪ Care-recipients benefitted from social interaction and meaningful activity with resultant improved well-being ▪ Carers wanted ADS service provision to meet the physical, mental and emotional needs of their care recipients	xxxx
Neville, Beattie, Fielding and MacAndrew (2015)	To examine the factors that promote the use of respite by carers of people with dementia.	N/A	Narrative review of (*N* = 76) studies Period covered was 1990–2012, but earlier articles were also reviewed.	▪ Utilization of respite services by carers of people with dementia is low ▪ The key topics identified were information access, barriers to carers utilizing respite, service satisfaction	xxxx
Logsdon, Pike, Korte and Goehring (2016)	To evaluate participant and caregiver outcomes of a dementia-specific program at an ADS.	(*N* = 187) participant–caregiver dyads i.e., (*N* = 162 attended ADS and (*N* = 25) comparison dyads who met the criteria for inclusion but did not attend an ADS	Mixed-method design	▪ Attendees exhibited a decrease in depressive behaviors ▪ Caregivers reported fewer stressors when compared to carers whose care recipients did not attend an ADS ▪ Wives using ADS placed their care recipients in nursing homes earlier than wives who did not use ADS and earlier than adult child caregivers who used the services ▪ Adult child caregivers reported better outcomes on measures of burden, distress, and depression than spousal caregivers ▪ This suggests that there may be important differences in spouse versus adult child caregivers in terms of ADS use and benefits	xxxx
Kelly, Puurveen and Gill (2016)	To compare delays to institutionalization between older adults who differed in the number of days they attended an ADS	(*N* = 16,012)	Quantitative research design using a Kaplan-Meier survival analysis and a Cox regression model	▪ Provides evidence for the beneficial effects of ADS attendance ▪ Risk of early institutionalization was reduced for attendees who increased the number of days they attended an ADS	xxxx
Tretteteig, Vatne and Rokstad (2016)	To explore the influence of ADS on family caregivers.	N/A	An integrative review of (*N* = 19) studies	▪ ADS provided respite service and support to family caregivers improving their ability to care for their care recipient ▪ The quality ADS provision was a predictor of its utilization	xxxx
Hynes, Field, Ledgerd, Swinson, Wenborn, di Bona, Moniz-Cook, Poland and Orrell (2016)	To explore how to make Community Occupational Therapy in Dementia (COTiD) relevant to the UK context.	(*N* = 39)	Qualitative study design utilising focus groups	▪ Issues such as early intervention and follow-up were emphasized ▪ Flexibility in service provision and programs that met the needs of people with dementia were motivating factors for attendance	xxxx

Number of X’s = the number of mixed-methods appraisal tool (MMAT) criteria met (Pluye et al., 2011).
